# Practical psychosocial care for providers of pre-hospital care: a summary of the report ‘valuing staff, valuing patients’

**DOI:** 10.1186/s13049-023-01141-6

**Published:** 2023-11-10

**Authors:** Richard Williams, Verity Kemp, Jennifer Burgess, Esther Murray, Suzy Stokes, Andrew Wood, Samantha Batt-Rawden, Laura Bland, David Lockey

**Affiliations:** 1 Welsh Institute for Health and Social Care, Faculty of Pre-Hospital Care, University of South Wales, Edinburgh, UK; 2 Director of the Psychosocial Care and Mental Healthcare Programme for the Faculty of Pre-Hospital Care, Royal College of Surgeons of Edinburgh 2018-2022, Edinburgh, UK; 3 Psychosocial Care and Mental Healthcare Programme for the Faculty of Pre-Hospital Care, Royal College of Surgeons of Edinburgh 2018-2022, Edinburgh, UK; 4 Cumbria, Northumberland, Tyne and Wear NHS Trust and Newcastle University, Newcastle Upon Tyne, UK; 5 Institute for Health Sciences Education, Faculty of Medicine and Dentistry, Queen Mary University of London, London, UK; 6 Emergency Medicine and Pre-Hospital Emergency Medicine, Oxford University Hospitals and Thames Valley Air Ambulance, Stokenchurch, UK; 7 Anaesthesia and Pre-Hospital Emergency Medicine, Barts Health NHS Trust, London, UK; 8 Intensive Care Medicine and Pre-Hospital Emergency Medicine, Ashford and St Peter’s NHS Foundation Trust, Cambridge, UK; 9 Pre-Hospital and Emergency Medicine, Somerset Foundation Trust and Dorset and Somerset Air Ambulance, Henstridge, UK; 10 Faculty of Pre-Hospital Care, Intercollegiate Board for Training in Pre-Hospital Emergency Medicine, Edinburgh, UK; 11 Faculty of Pre-Hospital Care, Royal College of Surgeons of Edinburgh, Edinburgh, UK

**Keywords:** Pre-hospital emergency medicine, Trainees, Systematic review, Secondary stressors, Wellbeing, Psychosocial needs, Mental health, Model of care

## Abstract

**Background:**

Caring for people who are ill or injured in pre-hospital environments is emotionally draining and physically demanding. This article focuses on the Psychosocial and Mental Health Programme commissioned by the Faculty of Pre-Hospital Care (FPHC) at the Royal College of Surgeons of Edinburgh (RCSEd) in 2018 to investigate the experiences and needs of responders to pre-hospital emergencies and make recommendations. It summarises the report to FPHC published in 2022, and adds material from research published subsequently.

**Method:**

FPHC appointed a team to undertake the work. Team members conducted a literature review, and a systematic review of the literature concerning the impacts on the mental health of pre-hospital practitioners. They conducted fieldwork, participated in training and had conversations with trainees and established practitioners, and took evidence from the Pre-hospital Emergency Medicine Trainees Association (PHEMTA).

**Results:**

The Results summarise the evidence-based theoretical background derived from the programme and practical guidance for practitioners, professional organisations, and employers who deliver pre-hospital care on the implications of, preventing and intervening with pre-hospital providers who experience psychosocial and mental health problems.

**Conclusion:**

This paper summarises the outputs from a multidisciplinary programme of scholarship, research, and fieldwork. The authors condense the findings and the guidance developed by the Programme Team to provide a summary of the report and guidance on implementation. They believe that the recommendations are applicable to all healthcare organisations and particularly those that employ responders to emergencies and provide pre-hospital care.

**Supplementary Information:**

The online version contains supplementary material available at 10.1186/s13049-023-01141-6.

## Background

The staff of health services are renowned for their resourcefulness under pressure [1–3]. Public expectations are that staff consistently deliver effective, evidence-based care and interventions compassionately even if their work environments are not optimal. However, it is difficult for healthcare staff to continue to provide compassionate, evidence- and values-based care for their patients without the support of their employers or if there is dissonance between the quality of support and training for staff and the quality of care that they are expected to deliver. This is especially so when employers implicitly or explicitly expect staff to take more than minor risks. This matter raises the issue of each organisation’s moral architecture [4]. Further evidence to support the findings and recommenations made in this paper and its relevance to supporting staff of all healthcare services comes from our work on the the COVID-19 pandemic and the Manchester Arena bombing [5–8]. Those experiences provide many practical lessons for caring effectively for healthcare staff and reducing the risks that they face.

Faced with pressures to deliver healthcare services in challenging circumstances, there is potential for practitioners to neglect their own physical and emotional needs. More extreme effects of exposing staff to crises and people’s suffering include burnout, compassion fatigue, and vicarious or secondary traumatisation. Much more common ones are distress and the COVID-19 pandemic illustrated just how common it is for staff to experience moral distress and moral injury [9].

The relationships between leaders, managers and clinical staff have been identified as predictors of both wellbeing and staff absence. Other factors include provision of sufficient resources, peer support, adequate information about events and tasks, and ensuring effective managerial and clinical supervision.

### The programme reported in this paper

Many surveys and reports demonstrate that the human cost of distress and mental health problems experienced by healthcare staff is huge and extends to their colleagues and families. Practitioners who work in pre-hospital emergency medicine (PHEM) are susceptible to these impacts. In 2018, the Faculty of Pre-Hospital Care (FPHC) at the Royal College of Surgeons of Edinburgh (RCSEd) established a programme to develop guidance on psychosocial care and mental healthcare for practitioners of pre-hospital care. The Faculty recognised that practical support was not always available to those who need it and that practical, safe support which was, as far as possible, evidence based needed to be accessible. This would enable pre-hospital organisations to put in place simple interventions to provide the necessary support and, where necessary, signposting to more specialised help.

A multi-professional working group was established, led by a psychiatrist who was an adviser on psychosocial care to NHS England and the Royal College of Psychiatrists (RW). The programme’s report advised on providing support for pre-hospital care practitioners, trainees, trainers, relevant professional bodies and employers by offering guidance on improving the care and support for practitioners. The full report, which was aimed specifically at UK pre-hospital emergency medicine, is on the Faculty of Pre-hospital Care website [10].

Colleagues have advised that the report’s recommendations are also applicable to healthcare workers affected by the pandemic and other events and to those who work in many other areas of care in many countries. Therefore, the authors have prepared this paper to summarise key findings and recommendations from the programme. It adds summaries of, and references to further developments in the topic area since the report was published in 2022. Advice on practical actions that may help when people identify concerns for themselves or their colleagues are summarised in the Additional file 1 to this paper.

## Method

### The programme team

FPHC in the RCSEd appointed a Director (RW) and Project Manager (VK) for the Psychosocial and Mental Health Programme and a number of experienced PHEM practitioners, trainees and mental health experts to the Programme Team (the Team). In the course of its work, the Team drew on and refined existing literature to address the context [11–13].

### Working methods

The Team adopted three main methods of work. This paper includes a brief summary here and readers are referred to the report for more detail [10].

First, the Team reviewed the literature and the outputs are informed by reviews of the available evidence from current clinical, scientific, managerial and policy sources. Summaries of key topics selected by the Team are included in the Results section.

Second, the Team conducted a systematic review of the literature about the impacts on the mental health of PHEM practitioners to identify the nature and scale of the recent challenges to their mental health. The Team aimed to describe current knowledge of the psychiatric and psychosocial consequences of working in pre-hospital care, and to identify any factors that could be causative or contribute to these impacts. The review was conducted according to the PRISMA guidelines and registered with PROSPERO (see: www.crd.york.ac.uk/PROSPERO/display_record.php?RecordID=157165). A full paper is being prepared and a summary has been included in a forthcoming book [14].

Third, members of the Team conducted extensive fieldwork by visiting practitioners and services, attending conferences and training events. The Team had conversations with many trainees and established practitioners at these events. A PHEM training programme was introduced in the UK in 2012. The Team participated in Introduction and Phase 2 Training Courses for trainees in Pre-Hospital Emergency Medicine in the UK organised by the Intercollegiate Board for Training in Pre-Hospital Emergency Medicine (IBTPHEM). As part of its fieldwork, the team took evidence from the Pre-hospital Emergency Medicine Trainees Association (PHEMTA). Surveys of PHEMTA members indicate that there are many positive themes in the experiences of doctors training in pre-hospital care. Usually, they enjoy their placements, feel well resourced, supervised, and supported at work. They also feel positive about the quality of care they deliver and recognise the positive impact this has on their patients and families. Nonetheless, those surveys also identify persisting problems that adversely affect trainees’ experiences. Many of those matters are secondary stressors.

### Consensus

As the Team completed its collation of evidence, it gathered in a series of face-to-face and online meetings to sift the evidence with which it had been presented. The Team wrote up its findings and ran a second set of consensus meetings to agree potential interventions, which led to it formulating recommendations. A draft report was circulated within the Team and adjusted until consensus on its contents was reached.

### Approval of the report

The penultimate draft of the report was presented to the officers of FPHC in RCSEd. Subsequently, the officers took the final version of the report to the Faculty’s Executive Committee for acceptance. The report was published in 2022.

## The results

This section summarises the Team’s findings and recommendations to the FPHC in four parts:

1. Items from the literature that are highly relevant to the report
2. A summary of findings from the systematic literature review
3. Problems most affecting PHEM practitioners identified by the Teams’ interviews with them
4. Responding to the needs of practitioners.

### Important items from the literature

The authors summarise important items drawn from the literature.

#### Terminology

The literature has documented the poor self-reported mental health of emergency service workers in the UK [15, 16]. However the terminology is a challenge because the phrase ‘mental health’ has a wide variety of overlapping meanings and uses. The term ‘mental health problems’ is used often but is imprecise; defining the terms used is important if we are to compare research results and offer the most appropriate forms of care. The terms used here and in the Additional file 1 are those in the glossary to the Team’s report. [10]

### Wellbeing

The WHO definition of mental health is ‘a state of well-being in which … [a person] realizes his or her own abilities, can cope with the normal stresses of life, can work productively and fruitfully, and is able to make a contribution to his or her community’ [17]. Thus, wellbeing refers to employees’ needs for sources of support to ensure that they are able to continue to develop, enjoy the stimulation of their work, and flourish.

### Psychosocial care

The construct of psychosocial care is based on taking a non-pathologising approach to meeting the needs of staff who are struggling or distressed but do not have a mental health disorder. They are likely to recover with support from their families and colleagues. Most people benefit from non-medical interventions that are based on the principles of psychological first aid (PFA) [18].

### Mental healthcare

Some staff may have more persistent needs and a small number may have problems that go beyond psychosocial care. They may have diagnosable mental health disorders and require skilled mental health assessments by general practitioners, occupational health teams and specialist psychiatric services.

#### Work, workplaces and mental health

In 2017, the Stevenson/Farmer review of mental health and employers reported that the human cost of mental health problems is huge, highlights higher rates of poor mental health and suicide for employees in healthcare services and recommends fostering and supporting good mental health in workplaces [19]. NHS Employers reported that, compared to people working in other professions, doctors are twice as likely and nurses are four times as likely to take their own lives to kill themselves [15].

**Fig. 1:**
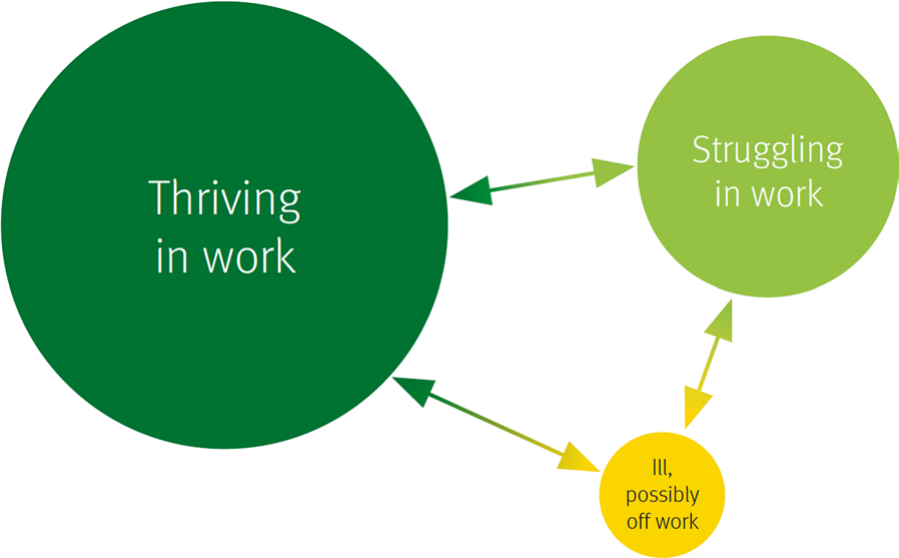
Three phases people experience in work (reproduced from Stevenson and Farmer [19], under Open Government Licence v3.0.)

The Stephenson/Farmer review identifies three broad groups of staff (Fig. 1) and considers how employees might be better supported.

By inference, the report presents three main challenges to employers and they are:

•Assisting employees to thrive at work
•Supporting staff who are struggling
•Enabling people who are ill to recover and return to work.

This advice resonates with our use of terminology and led to the Team agreeing the three interlinked objectives for caring for staff shown in Table 1 [5]

**Table 1:** Three agendas in caring for staff

The wellbeing agenda	Assisting people to thrive at home, in work or at school*.* Wellbeing is about feeling good and functioning well and is influenced by each person’s experience of life
The psychosocial agenda	Supporting people who are struggling. Psychosocial care describes interventions for people who are distressed or struggling or have symptoms of mental health problems that do not reach a diagnosis whether or not they also suffer social or work dysfunction
The mental health agenda	Enabling people whose needs appear to go beyond struggling to access mental healthcare for timely assessment and, if necessary, treatment and support with recovery and returning to work or school

#### Families

Research on survivors of the Manchester Arena bombing in 2017 shows just how important is support from families and support from other people involved in the same emergency to survivors’ early coping and recovery [6–8]. Social support and contributing to community development, which are both parts of PFA, can accelerate survivors’ longer-term adaptation and recovery and aid prevention of their developing mental health disorders in the medium- and longer-terms. Evidence from UK research with family members of firefighters showed that families/relatives [20]:

•Have a strong need for their sacrifices to be recognised
•Avoid engaging with the perceived occupational risk of their family members instead trusting in their training, equipment, and colleagues
•Provide a shared identity and support network
•Often try to undertake ongoing assessments of their relative/family member to calibrate health and wellbeing.

Healthcare staff may be hesitant to discuss at home the details and emotional impact of their work in order to protect their families despite the evidence suggesting that families can help to alleviate worries by encouraging them to talk about their jobs and what they entail. Recently, we have seen recognition of the impact of the changes to work patterns created by the COVID-19 pandemic [5].

#### Primary and secondary stressors

Emergency responders are a mix of people from several disciplines and agencies who have differing capabilities, roles and experiences. They face differing profiles of psychosocial risk and needs for education, training, and social and peer support. Most pre-hospital workers in the UK are employed by ambulance services. A smaller group of clinicians, which includes nurses, paramedics and doctors, work in highly specialised pre-hospital critical care units, and respond to high acuity incidents. These units are often well-resourced and surveys have emphasised the positive aspects of this working environment.

PHEM practitioners are exposed to, and witness suffering, distress, and death, with unusually high frequency and are under particular pressure due to the increased and increasing demand on their services. These factors are known as primary stressors. They are the sources of worry, anxiety or pressure that stem directly from the events and consequential tasks that the staff face in delivering high-quality care for patients. They may reflect single events but, more often, are an accumulation of pressure over time. Research suggests that levels and duration of exposure are a major risk factor for people who develop mental health problems, such as symptoms of PTSD [21], and this isoften referred to as ‘the dose effect’. Much more recently, academic studies of the impacts of being exposed to multiple adverse events have been published [22, 23]. The cumulative and interactive effects may persist over lengthy periods and challenge the habituation fallacy [24]. The clinical workloads experienced by PHEM practitioners may be challenging cognitively, emotionally, and psychosocially. However, presumptions of positive adaptation have allowed a myth, the Habituation Fallacy, to arise and the truth is that repeated exposure to severe adversity makes it harder, not easier, for disaster survivors to cope with a new negative event [24]. This is an additional reason for focusing on meeting the needs of staff who work in challenging environments and experience recurrent exposures to emergencies.

The work of PHEM practitioners is likely to be stressful by virtue not only of the enormity of other people’s suffering (primary stressors) but also the demands of performing demanding and skilful work in hostile environments. The latter pose secondary stressors. PHEM is an arena of specialist practice where potent primary and secondary stressors meet. The stress experienced in pre-hospital environments is influenced by social factors, life and work circumstances (including policies, practices, and social, organisational, and financial arrangements) and societal and organisational responses to an incident or emergency [25].

Research into children’s development suggests that ‘… long-term adverse outcomes are better predicted by the total number rather than the specific nature of environmental risk exposures’ [26]. We think this applies to adults too. Secondary stressors and adverse outcomes can be modified by the adequacy and effectiveness of employers’ responses to events and expectations of employees’ performance, career aspirations, and concerns of staff about their training, the conditions in which they work and live and their work-life balance. Secondary stressors can persist for longer than most emergencies and can limit recovery and adaptation. The work that underpins this paper has suggested that secondary stressors have a greater potential to affect pre-hospital workers than do primary stressors and the matters raised by staff who seek support frequently concern secondary stressors.

## Findings from the systematic literature review

The systematic review established that considerable interest has been directed towards concerns that: [14]

… practitioners who work in pre-hospital care may develop burnout and psychiatric disorders, in particular, and that they may develop post-traumatic stress disorder as a result of attending critical incidents. However, the methods used by most of the studies in our sample were not able to answer … questions [about the frequency of diagnoses and other conditions] because they used cross-sectional surveys with convenience samples and self-report questionnaires, which are not diagnostic tools. They considerably over-estimate the incidence of these problems, as demonstrated by the one high quality study which conducted clinical interviews and found that … a small[er] percentage of employees met criteria for PTSD or major depression, and that most resolved over a few months.

However, the high scores on these questionnaires probably indicate that PHEM practitioners often suffer considerable stress and distress. The sources of this stress are not as likely to be, as has often been thought, attending unusual and perhaps high-profile incidents, but more related to daily organisational and operational hassles such as unsupportive managers and a high volume of work to be done despite lack of resources.

### Problems most affecting PHEM practitioners identified during the teams’ interviews with them

The Team found that a number of conditions that were commonly reported by practitioners and pre-hospital emergency trainees.

#### Distress

Distress is the most common impact of working in pre-hospital care settings. Distress is not a disorder but may accompany disorders. Some of the literature refers to distress being comprised of symptoms of anxiety, depression, or post-traumatic stress disorder. Most people report symptoms on self-completed questionnaires that do not reach cut-off levels that might indicate that they should be assessed to determine if a diagnosis of a common mental disorder is indicated on clinical grounds. Another approach involves considering the range and severity of people’s experiences against a checklist of common experiences that have been reported in previous incidents. Often the range of experiences considered is broader than the symptoms of common mental disorders [6, 8, 10]. Perhaps the most practical approach is based on people’s subjective descriptions of what they have experienced. A useful definition of distress during and after emergencies is based on the observation that [6, 8]:

People are likely to feel stressed in emergencies and incidents. Their experiences are described as distress when they are accompanied by emotions, thoughts, and physical sensations that are upsetting or which effect their relationships. Recent research shows that common experiences that people describe as distress include feeling upset; fear; anxiety; fear of recurrence of the event; vigilance at social gatherings and in public places; avoiding uncomfortable feelings; and social withdrawal [6, 8]. The main differences between distress and the symptoms of common mental health problems lies in the trajectory of people’s recovery and the severity of their experiences. Until recently, the literature has tended to underestimate the number of people who take a long time to recover.

Practical aspects of these matters are illustrated by four papers [5–8].

#### Fatigue

Three main sources of fatigue are [10, 27]:

•Working at unfavourable times of the day (the circadian factor)
•Being short of sleep before starting work and/or prolonged prior wakefulness (the homeostatic factor) and
•Task-related factors (the physical and mental task demands).

Shift working in pre-hospital emergency care can cause disturbances in people’s natural sleep–wake cycles and disrupt circadian rhythms. Shift-workers typically accrue a *sleep debt* as sleep is reduced in both quantity and quality and sequential night shifts compound risk. Therefore, it is not surprising that pre-hospital care providers consistently describe high levels of fatigue. Job cycles can be lengthy and can increase fatigue, even when people are well-rested prior to a mission. Shift-work and fatigue carry a significant psychological morbidity.

#### Secondary stressors

There are many secondary stressors in addition to primary stressors experienced by PHEM practitioners whether trainees or established trained practitioners. The Team found that secondary stressors are prominent in pre-hospital emergency work—see Table 2.

**Table 2:** Examples of secondary stressors reported by PHEM trainees

Risks arising from responding using emergency vehicles or airframes	Inadequate skills or training to do the job
On-scene dangers	Inadequate equipment needed to do the job
Fatigue due to shift and night work	Poor role definitions and unclear expectations
Long commutes after work	Unnecessarily poor working conditions
Separation from friends or family support due to workplaces being distant	Conflict and mistrust within or between teams

#### Moral distress and moral injury

The concept of moral distress was outlined by Jameton in 1984 [28]. It refers to the effects of knowing what should be done for a patient but being unable to do so because of situational and organisational constraints including lack of time, staff or equipment.

Moral injury has been described as the betrayal of what is right by someone who holds legitimate authority, in a high stakes situation [29], and as the result of: ‘perpetrating, failing to prevent, bearing witness to or learning about acts that transgress deeply held moral beliefs or expectations’ [30].

After morally injurious events, the experiences tend to revolve around shame and guilt, with concomitant withdrawal from social networks and isolation. Cognitive models of PTSD conceptualise symptoms as the result of the interactions of the mind with extreme fear in which the sufferer appraises the world as an unsafe place in which terrible things can happen. By contrast, the concept of moral injury suggests that the mechanism of action might be more closely related to feelings and thoughts about shame and guilt, that is, the world is a *wrong* place, in which terrible things are *allowed* to happen. Researchers believe that the guilt and shame tend not to reduce over time unless emotions are effectively processed [30, 31].

PHEM has a strong tradition of regular debriefing, flat hierarchies and teamwork, which may go some way to mitigating the effects of moral distress and moral injury, as does good leadership. The distress, dysfunction and disorders that staff experience are similar to the conditions that affect survivors of incidents and emergencies. Staff who experience distress that persists for more than two weeks require assessment.

#### Caring responsibilities and parental leave

Although less than full time training has become more established, arranging childcare around long shifts, at antisocial hours, and with unpredictable finish times can be sources of significant stress and fatigue.

#### Burnout

The systematic literature review showed that the most used scale was the Maslach Burnout Inventory [32]. Burnout is not a medical condition but a syndrome of chronic workplace stress and reflects a process that runs from high expectation and idealism to irreversible loss of interest and personal breakdown’ [32]. A recent guide describes burnout as ‘a state of physical and emotional exhaustion due to excessive and prolonged interpersonal work-related stressors’ [33]. It has three dimensions: emotional exhaustion; depersonalisation or cynicism; and reduced professional efficacy [34]. Distress, fatigue and moral distress that are experienced over substantial periods by practitioners plainly create risks of their becoming burned out.

### Responding to the needs of practitioners

#### Initial responses to staff who are distressed or at risk of being adversely affected by their exposure to emergencies

It is important to recognise that stress and distress are common reactions and not usually indicative of pathology though they may accompany mental health disorders. That is probably because working in small teams over long shifts provides the opportunity for natural conversations and peer support. It is important to develop a culture in which people feel valued and safe and can form helpful relationships with their colleagues. This emphasises the importance of having psychosocially-informed conversations embedded within organisations’ cultures.

Most pre-hospital emergency organisations care about employees’ wellbeing and are supportive. Many have rigorous governance processes in which cases are scrutinised in a systematic way. This often involves a technical debrief and discussion of cases in detail and is often highly valued education. However this type of reflection can create situations in which clinicians are expected to recount events and to re-live difficult or distressing events in front of peers, colleagues and supervisors. There is evidence that ‘debriefing’ of this nature has the potential to cause harm and that it should be avoided. Therefore, it is important to understand and select cases for open peer review sensitively. The UK’s National Institute for Health and Care Excellence (NICE) states that psychologically focused debriefing should not be offered for preventing or treating PTSD [35]. However, this is hugely different to teams sensitively offering mutual support and conducting emotional discussions. The Team’s experience while conducting the review was that many PHEM teams handle this sensitively and constructively. This process is described in a recent resource as an Operational Debrief conducted within the responding team in which opportunities may be taken to emotionally support members [36]. Research has shown that the three items covered next aid this approach [6–8]. Recommendations in a recent book and papers offer further support for the approaches recommended here [37–44].

### Validation

People who are affected by emergencies and incidents regard social and professional acknowledgement of their experiences as key to their recovery. This process is called validation and describes recognition or affirmation of distress. Often colleagues and family members are the most important sources of validation. Validation by a professional person confers positive connotations on a person’s distress, their wish to seek support and may establish entitlement to care offered by a person perceived to have particular knowledge of the psychosocial impacts of major events. Validation challenges negative self-evaluation. Research on the Manchester Arena bombing has confirmed it as an important component of the initial approach to supporting people whether survivors or responders [5–8].

### Listening

Active listening (making a conscious and trained effort to hear not only words but the complete message being communicated) is core to helping to support the wellbeing of colleagues.

### Leadership

Leaders have a core role in addressing the impacts of stress on the workforce of their organisations. They should also be mindful of their own needs because responsibilities for other people are acknowledged to bring additional stress. Leaders should create a culture of safety, both from systems (aviation and clinical in PHEM) but also emotionally (it is okay to speak up, and admit fears, weaknesses, errors, and uncertainty and to express emotion). There is evidence that creating a culture of psychological safety reduces errors [45]. Leaders should also be familiar with key concepts relating to psychosocial care and shaping the culture of teams and environments so that teams are psychosocially informed and safe. Leaders should lead by example (e.g., by sharing learning from mistakes they have made and being open about their weaknesses). Organisations have a responsibility to ensure that staff can access support when they are concerned about their wellbeing.

#### A programme for support and care based on 15 key approaches

Healthcare staff who work in pre-hospital environments are required to do demanding and skilful work in hazardous environments. They are exposed to extraordinary events and may witness suffering, distress and death, with unusually high frequency. Inevitably, some of the impacts are stressful. The Team concluded that there are 15 key approaches for all organisations in their care for the wellbeing, psychosocial and mental health needs of their PHEM staff that are summarised in Table 3. There is more detail on the activities that assist staff in the Additional file 1.

**Table 3:** 15 key approaches for organisations that employ PHEM practitioners

	Provide clear messages about the priorities of work and care for staff within organisations
	Ensure every employee has a person or a place to which they can go for immediate support and ensure staff have space and time for reflection
	Ensure that work is based on effective teams and that team cohesion is supported by employees training together
	Ensure that leaders are effective and supportive to enable people and to develop team cohesion
	Develop care pathways that link the wellbeing, psychosocial and mental health aspects of the organisations’ workforce support plans
	Intervene early with staff who are distressed; this requires strengthening the working environment, and listening rather than initially providing therapy or counselling
	Adopt a practical approach to early intervention based on the acronym PIES; that is providing interventions in proximity to where people work, with immediacy and expectation of recovery and by using simple interventions first. There is evidence that this approach lessens the risks of staff members developing mental health disorders later
	Yse active listening skills
	Seek out and remedy secondary stressors
	Ensure that employees are offered opportunities for integrationg with their peers because social support is key
	Remember that colleagues’ sustaining their senses of personal efficacy are important in their recovery
	Consider setting up peer support programmes because they bring staff in departments and teams together and may prevent development of more serious problems [46]
	Be clear about who will and will not benefit from a ‘medical’ approach (a minority of people may develop diagnosable mental health disorders for which they require specialised medical care, but most do not)
	Support staff in the face of negative public perceptions
	The actions in this list are all critical to creating environments at work that are conducive to staff giving of their best. Policies and actions for supporting staff must be separate from those for staff discipline and performance management

#### A practical commentary on the key approaches

Pre-Hospital Employers Should Reduce Primary and Secondary Stressors.

The effect of primary stressors, which tend to receive the greatest attention in practice and research, may be reduced by adequate preparation, training and supervision. However, experience and research show that secondary stressors are not only potent but also frequent and often amenable to improvement.

Although primary stressors are very powerful in pre-hospital working environments, there are also many sources of secondary stressors. There may be a tendency to consider them of lesser importance but that would be a serious error because secondary stressors may be more impactful causes of problems for staff and an active plan is required to remove them or mitigate their effects. Employing organisations should take steps to identify and mitigate the secondary stressors experienced by their staff.

The Resuscitation UK Resuscitation Council UK (RCUK) considers it a duty to prepare all responders for the possible negative impact of a resuscitation event on their mental health and wellbeing [36]. It offers a video, an online resource for all responders and makes recommendations about a post-resuscitation procedure. In similar terms, an online teaching programme for Blue Light Services identifies how the principles in this paper might be put into effect (available at: mindedhub.org.uk/media/quvlpqkv/minded_brochure_a4_r6.pdf) [47].

### Cohesion and leadership are vital to good care of staff

There is copious research to support conclusions that working in well-led, coherent teams is an important contribution to getting right the culture of health and social care organisations and is likely to offer strong protection for the staff wellbeing. This means being clear about: the nature of leadership that is required; the importance of being offered a buddy or mentor; access to a place and person to which staff can go if they are stressed; and the importance of supporting peer groups.

Often, the problems that affect staff of PHEM services are not indications that staff have developed or are developing mental disorders. This reveals the problem with terminology and the huge potential for misunderstandings about the meaning of terms such as welfare, wellbeing, psychosocial care, and mental healthcare. This confusion contributes to people’s reluctance to accept support and to stigma.

We recommend that the main firstline approach to caring for staff should be non-medical, which should be made readily available. Everyone should have access to facilities that are able to support staff in flourishing and gaining satisfaction and positive experiences from their work. A number of staff may be distressed by their experiences at work or the conditions in which they work.

However, a small proportion of staff may develop mental health problems of more serious natures that may require evidence-based, specialist assessment and treatment. There should be no complacency about this, and the non-medical and non-specialised facilities that offer psychosocial care should be capable of signposting of people in need to more specialised services as early as possible usually through occupational health services or primary care. It is only when staff are thought to need mental healthcare for a diagnosable disorder that their circumstances should be medicalised.

### A stepped approach to care of staff

Increasing numbers of papers make recommendations for how employers should organise the responses to the needs of their employees including those who deliver PHEM [2, 11, 13, 48]. The authors’ opinion is that this should consist of: a universal wellbeing agenda for everyone; focused psychosocial care for those people who are struggling and/or distressed that can be used without formal referral; and agreed pathways for people who need, or appear to need specialist mental healthcare. Based on the Stevenson/Farmer review of mental health [19], the Team recommends that employers foster and support good mental health by attending to the three challenges covered in Table 4 [5].

**Table 4:** The three challenges

The wellbeing agenda	Assisting employees to thrive at work*.* Wellbeing is about feeling good and functioning well and is influenced by each person’s experience of life. In practical terms organisations should provide:
	Interventions to sustain the wellbeing of members who are thriving and enable them to move on towards flourishing through engaging members in their own emotional and cognitive development
	A programme of workplace development that:
	Is informed by awareness of the kinds of primary and secondary stressors that members face
	Endeavours to reduce the primary stressors to a minimum
	Responds to and remedies the secondary stressors that impact member
	A plan for developing teams and teamwork and integrating personal, team and workplace support programmes
	Recognition of the nature and impacts of secondary stressors and reducing their impacts on members
	Ease of access for members who may have more serious and persistent problems to specialised mental healthcare
The psychosocial agenda	Supporting staff who are struggling
	The distress that staff experience and the dysfunction and disorders they risk are similar to the conditions that affect survivors of significant and major incidents
	Yet, staff may feel stigmatised by recognising or showing the emotions they experience and any problems they develop. Staff who experience distress that persists for more than two weeks after a significant event should receive assessments of their needs
	Psychosocial care describes interventions for people who are distressed or struggling or have symptoms of mental health problems that do not reach a diagnosis whether or not they also suffer social or work dysfunction. This includes encouraging departments to create peer support programmes for members who are struggling
The mental health agenda	Enabling people whose needs appear to go beyond struggling to access mental healthcare, recover and return to work
	Employers may need to negotiate service level agreements with mental health providers

### Strategic underpinning for a stepped programme of care for PHEM staff

Table 5 summarises a number of actions that employers should take in order to underpin the approach recommended in this paper.

**Table 5:** Actions to support their strategy that employers should consider include

	Develop a strategy for supporting the wellbeing, psychosocial care and mental health of their staff. Staff should be aware of the existence of this strategy and should have access to it
	Review how pre-hospital trainees are selected and allocated to placements with a view to reducing secondary stressors
	Address the expected working patterns and geographical locations of trainees and working sites to minimise secondary stresses which result from long hours, long commutes, separation from friends and families and disruption of carer responsibilities
	Promote research to gain knowledge of the scale and impact of the exposure of their staff to distress arising from their work
	Promote awareness of the emotional labour ordinarily carried out by their staff and of ways to cope with it. A substantial amount of emotional labour is implicitly required by pre-hospital healthcare professionals who regularly support patients and their families through great suffering and the most distressing events
	Promote knowledge of the evidence showing that responders are likely to be at risk of the psychosocial and mental health consequences of their involvement in significant incidents
	Promote awareness of the evidence showing that employees gain psychosocial benefits from knowing that their employer has a strategy in place to support their psychosocial and mental health and that employees who are well supported tend to make fewer mistakes

## Discussion

This paper summarises the outputs of a substantial multidisciplinary programme. It was designed to provide an evidence-based, theoretical background and practical guidance for practitioners, including trainees and experienced practitioners, professional organisations, and employers that deliver pre-hospital care on the implications of, and preventing psychosocial and mental health problems experienced by pre-hospital providers.

The original report contains similar information and is freely available but was targeted at a specific UK pre-hospital audience [10]. This summary is modified to ensure that it is applicable to a wider range of healthcare organisations in the UK and elsewhere. It has been updated to signpost recent publications. The format is brief and outlines only the key elements of what is required. Some organisations already have comprehensive arrangements in place while others may have only recently started to consider what organisational infrastructure may be necessary.

The Additional file 1 provides: more information on the ‘Integrated Psychosocial Approach’ described in the recommendations; practical guides to ‘Dos and Don’ts in Caring for Staff’, which many organisations may find essential when providing support for troubled colleagues; and a section on ‘Psychological First Aid for Pre-hospital Practitioners.’ Finally, the file lists some online resources, and provides the glossary of terms used in the report.

Increasingly, the importance of staff wellbeing has been discussed as a core feature of highly performing organisations. The recent pandemic has highlighted the requirement for organisations to deliver appropriate support to their frontline staff. We hope that the material presented in the report [10], and this paper assists pre-hospital organisations to achieve these vital aims.

### Recommendations

The core of the guidance is condensed into a single table, Table 6, which summarises the report to the FPHC but has been adjusted to be appropriate to all emergency and other healthcare organisations.

**Table 6:** Recommendations for how employers should support and care for their healthcare staff

*Core principles*	
**1**	There is no health without mental health
**2**	The mental health of their staff is the core concern of all healthcare employers because the quality of care for patients depends on having healthy and effective staff. Employers and staff should champion actions that help to support the wellbeing, psychosocial care, and mental healthcare of staff. Clinical errors are reduced in such an environment
*Practical actions by employers*	
**3**	Healthcare employers should:
	a. Offer an accessible, stepped programme of wellbeing, psychosocial and mental health care for all staff who need it consisting of:
	i. Defined, universal and continuing support for staff wellbeing that is integral to job plans and the way in which organisations manage staff and conduct governance
	ii. Psychosocial interventions that are readily available, without referral, for staff who are struggling
	iii. Specialist mental health assessments and treatments that are available for staff who need them that enable staff to be referred rapidly for assessment and treatment when necessary
	b. Recognise the importance of secondary stressors (e.g., long commutes to and from work-bases when fatigued, unsatisfactory accommodation, and poor access to showers and hot food) and act to reduce them to a minimum
	c. Recognise the moral struggles that staff may experience in demanding situations and provide the help they may need to cope with the ethical challenges in their work
	d. Enable departments to create peer support programmes and employing trained mental health practitioners to offer supervision and support for peer supporters
	e. Integrate these arrangements into processes of workforce and workplace development, and emergency planning and preparedness
*Teams and leadership*	
**4**	Working in teams is integral to delivering high quality care for patients and promoting the mental health of staff
	a. This means that employers and senior staff should work to ensure all team members:
	i. Feel connected and supported by their colleagues
	ii. Have a buddy of their choosing
	iii. Work within teams that have stable relationships
	iv. Have well-functioning communications
	v. Are well-trained
	b. Where and when possible, team development should be supported by co-location of members, alignment of work schedules and enable team members to express their views about their preferences about with whom they work
	c. Employers and senior staff should recognise that teams are not merely groups of people but have shared identities. This requires substantial planning, preparedness, training, and support
	d. Working jointly in situations that need the combined and coordinated work of several agencies is often required of organisations that respond to incidents. Often, teams are composed of people from a range of agencies, each having its own structure and culture. This requires staff in emergency medicine and related specialties to work in effective ways across not only disciplinary but also organisational cultures. Training should enable staff to work harmoniously and effectively with staff in partner agencies
	e. Working in situations that require the combined and coordinated work of several agencies is often required of organisations that respond to incidents. Often, teams are composed of people from a range of agencies, each having its own structure and culture. This requires staff in emergency medicine and related specialties to work in effective ways across not only disciplinary but also organisational cultures. Training should enable staff to work harmoniously and effectively with staff in partner agencies
	f. Teams should be well-led by people who are selected for having appropriate skills and receive continuing support and training
	g. Leaders should be selected and trained in the process of emergency planning and preparedness
*Emergency planning and preparedness*	
**5**	a. Psychosocial care should be regarded as an essential part of emergency responses and recovery and, therefore, must be an equal consideration in planning. The process should include experienced planners, people with experience of working across agency boundaries and mental health specialists in advisory structures at all levels
	b. Psychosocial care should be regarded as an essential part of emergency responses and recovery and, therefore, must be an equal consideration in planning. The process should include experienced planners, people with experience of working across agency boundaries and mental health specialists in advisory structures at all levels
	c. Horizon scanning, assessment, and surveillance are tools to try to predict when unusual demands may occur that are likely to tax emergency planning and preparedness and frontline staff. Their use must extend to the potential psychosocial and mental health impacts of events on staff to enable their preparation to meet unusual demands
	d. Training in the requirements of the emergency plan should focus on the process of emergency planning rather than the exact nature of plans. Processes identified in every emergency plan must be rehearsed and tested in realistic exercises with people in key positions in host and partner organisations to enable them to build relationships and develop experience of effective engagement and interoperability

**Fig. 2:**
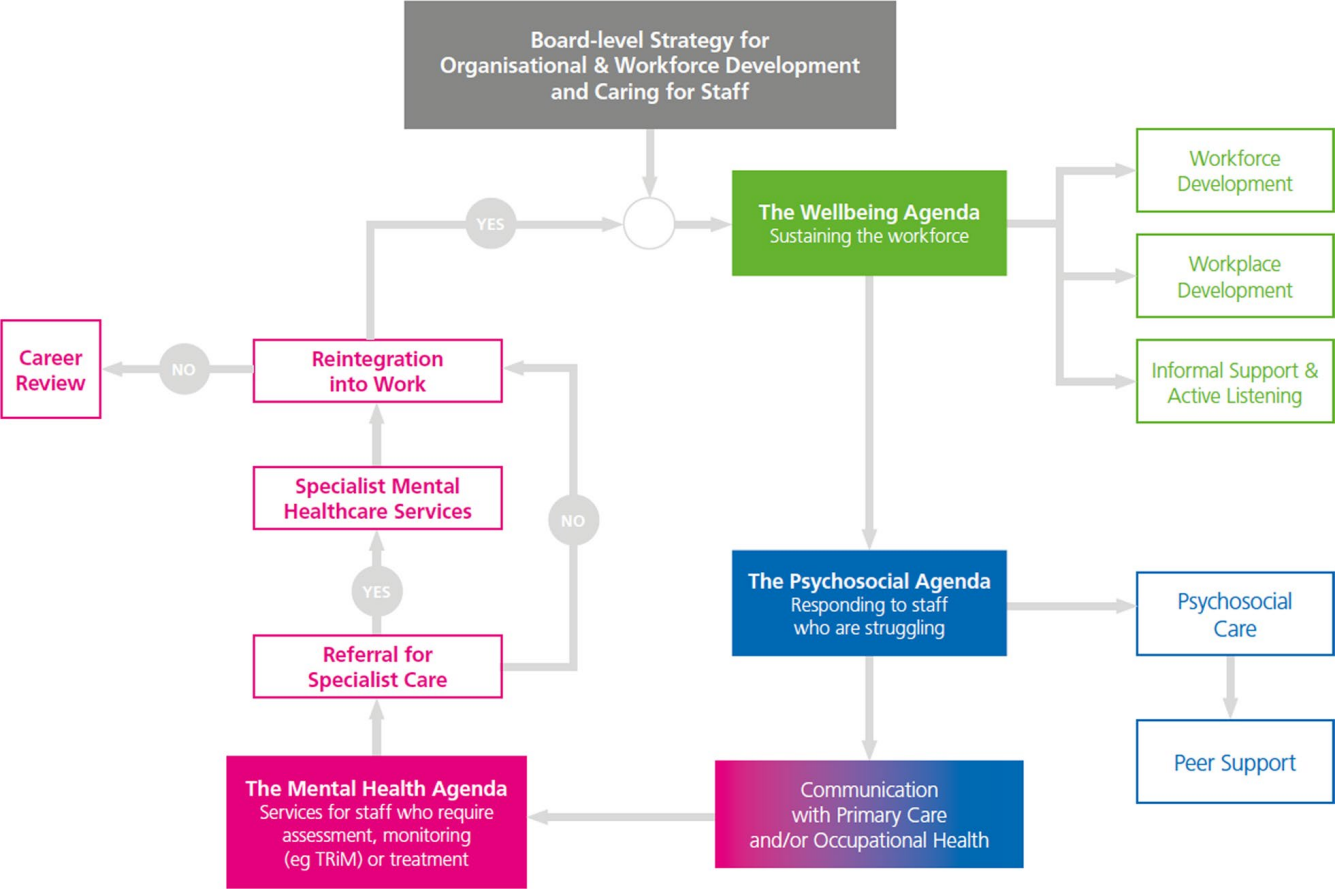
A model of care © R Williams, V Kemp, 2021 all rights reserved

This paper reproduces, as Fig. 2, the schematic diagram [10], that the Team created as a strategic summary of how it envisaged that organisations might create and link facilities and processes to develop and support their staff. This approach has been adopted by NHS England. [48].

## Conclusion

The contents of this paper and also the report on which it is based draw on established principles and their recent more detailed interpretation in the light of recent scientific developments as well as lessons gleaned from working through the COVID-19 pandemic. This paper and our recommendations are compatible with recent guidance from NHS England [48].

The authors are aware of the continuing developments in the topic area since their report was published in 2022. They believe that the importance of the wellbeing, psychosocial and mental health agendas has continued to rise not only in the UK but in many other countries. They hope that putting this work into the public domain assists other jurisdictions to progress their own work on protecting and caring for staff who deliver pre-hospital emergency medicine.

### Supplementary Information

**Additional file 1.** Supplementary material.
