# Supplementary material for: Practical psychosocial care for providers of pre-hospital care: a summary of the report ‘valuing staff, valuing patients’
Source: Scand J Trauma Resusc Emerg Med. 2023 Nov 10;31:77. doi: 10.1186/s13049-023-01141-6 (PMC10636848; doi:10.1186/s13049-023-01141-6)
Supplement: Supplementary file 1 — Additional file 1. Supplementary material. [file 13049_2023_1141_MOESM1_ESM.docx]

## SUPPLEMENTARY MATERIAL

## THE INTEGRATED PSYCHOSOCIAL APPROACH

This file is based on material developed for the report Valuing Staff, Valuing Patients, for the Faculty of Pre-hospital Care of the Royal College of Surgeons of Edinburgh.^1^

Williams and Kemp have created an Integrated Psychosocial Approach.^2,3^ Informed by Patel,^4^ it describes:

- Distinguishing people who are distressed from those who require biomedical assessments and treatments
- Making distinction between the two sorts of conditions on trajectories of people’s stress levels and dysfunction
- Providing assistance for most distressed people through lower intensity psychosocial care.

These recommendations are presented in a format that is consistent with the approach taken in the Stevenson-Farmer Review and takes account of the British Medical Association’s charter for employers.^5,6^ We recommend a strategic approach that includes these principles.

**IMPROVING CARE FOR STAFF**

The report proposes a framework that follows the categories identified in the Stevenson-Farmer Review and consists of a set of core principles and a model of care.^1^ That model has been taken up by NHS England. It identifies in a series of tools, methods for planning and caring for people, the principles of psychological first aid (PFA) and Dos and Don’ts in caring for staff, and how to recognise and manage stress and distress. The following section summarises those tools.

**PLANNING**

Planning is important and can sustain staff through an emergency or the declaration of a major incident. The three agendas we describe in our model apply both to unusual circumstances and ‘business as usual’. This model suggests core actions to support mental health and wellbeing in staff for these situations. Experience suggests that emergencies cause unusual primary stressors and accentuate the impacts of secondary stressors.

Psychosocial care is intended to sustain people in the aftermath of an incident or emergency, and reduce their distress, suffering and risk of developing a mental disorder. It should be available to anyone who is affected by an incident.

Mental healthcare should be available to anyone who has moderate to severe mental health needs, including, importantly, people who have a diagnosed mental health problem or disorder. These interventions are usually delivered by specialists who work in mental health services.

The psychosocial care and mental healthcare responses to incidents and emergencies must be well planned so that people can obtain the right help at the right time by ensuring the psychological safety of all people involved. Key elements of these responses should be underpinned by the provision of information for the public about people’s distressed reactions to incidents. This will support the ability to distinguish people who are distressed and require psychosocial care from people who also require mental healthcare. The offer of early intervention and active outreach will help minimise distress and reduce the numbers of people at risk of developing longer-term mental health problems.^7^ Lower-intensity psychosocial interventions should be available to the substantial numbers of staff who are likely to be distressed in conjunction with the timely provision of more specific interventions e.g., evidence based psychological interventions, for people assessed as needing these. This is all built on recognising the important role of GPs in identifying people in need , monitoring, and supporting people affected and recognising when and how to refer them to specialist services.

Planning should embrace staff and their families. It should establish the initial support to be delivered, based on providing clear and consistent routes of communications to all key stakeholders. As always, approached must be evidence based, proportionate, flexible, and timely. There should be well-defined multi agency response provided within a clear governance framework that supports co-ordinated delivery of care aimed at managing key interfaces and transitions seamlessly. Provision should be made by professional practitioners, managers and staff who are appropriately qualified and have access to training, good leadership, support, and supervision.

A journal paper reviews the impact of a four-hour training programme for commanders of firefighters in Australia showing that a four-hour mental health training programme for managers led to a significant reduction in work-related sickness leave and a return on investment of £10 for every pound spent on the training.^8^

Organisations and individuals should be aware that:

- A common but erroneous assumption is that everybody involved needs counselling or psychiatric treatment in the immediate aftermath of a major incident.
- Social support is a natural and powerful intervention.

Single-session stress debriefing and brief interventions that require people to re-experience the events that they have survived should not be practiced. In the UK the National Institute for Health and Care Excellence (NICE) states that psychologically focused debriefing should not be offered for preventing or treating PTSD.^7^

In summary, the psychosocial care and mental healthcare responses to incidents and emergencies must be well planned, timely, agile and characterised by a focus on the restoration of physical and psychosocial safety for people and their communities. They must be culturally appropriate and enable choice and control for people affected. Active outreach, evidence-based interventions and collaboration with NHS and non NHS providers will also be key features. This approach will enable the evaluation of outcomes and their cost effectiveness.

**CARING FOR STAFF AND OTHERS**

In this section, we summarise some of the practical tools that can be used to help support staff, their families, people and communities affected by the immediate and longer-term impacts of emergencies and incidents. All practical tools are evidence informed.

**PSYCHOLOGICAL FIRST AID (PFA) FOR PRE-HOSPITAL PRACTITIONERS**

PFA is one of the principle practical tools recommended. It is a well-established, evidence based, globally recommended method for supporting people during emergencies on delivering psychosocial care in the immediate aftermath of an event. It can be used to offer emotional support and coping strategies to support people affected by the on-going nature of their work.

At and after an emergency, it is likely that staff will be working with people, including colleagues, who are upset, fearful, worried and possibly confused, at least in the initial stages. They be apprehensive about the future and their recovery, sleep poorly and/or have nightmares. They may be anxious and have a loss of confidence in themselves and others. Later, they may be bereaved and grieving and especially so if someone close to them is injured or killed. It is important to remember that relatives and close friends may be having very similar experiences and may also need support.

The goal in providing psychological first aid is to promote an environment of safety, calm, connectedness, self-efficacy, empowerment, hope, healing, and confidence in their professional carers.

**The Dos and Don’ts in Caring for Staff**

This list of suggested of Dos and Don’ts in Caring for Staff are developed from a briefing paper for professional and general managers The Top Ten Messages for Supporting Healthcare Staff During the COVID-19 Pandemic published by Williams et al., in early 2020.^9^

*Things to Do*

1. Be Kind to Yourself and One Another.
2. Assist staff to Manage their Concerns.
3. Encourage Staff to Sustain Their Social Connections.
4. Respond to Moral Distress and Ethical Considerations.
5. Remember to Eat, Drink, Rest and Sustain Contacts with Friends and Take Breaks.
6. Continue Supervision and Relevant Training.
7. Challenge Incipient Loneliness.
8. Support for Frontline Staff Should be Visible.
9. Follow Assessment and Treatment Protocols.
10. Be Aware of authoritative advice and guidance as it emerges.

*Things Not to Do*

1. A common but erroneous assumption is that everybody involved needs counselling or psychiatric treatment in the immediate aftermath of a major or untoward incident or an emergency.
2. Avoid routine screening because there is little evidence that it conveys benefit inside organisations.
3. Single-session stress debriefing and brief interventions that ask people to re-experience the events that they have survived should be avoided. NICE states that psychologically focused debriefing should not be offered for preventing or treating PTSD.^7,10^
4. Force people to share their stories with you, especially very personal details.
5. Give simple reassurance like ‘everything will be OK’ or ‘at least you survived’.
6. Tell people what you think they should be feeling, thinking or how they should have acted earlier.
7. Tell people why you think they have suffered by alluding to personal behaviours or beliefs of people affected.
8. Make promises that may not be kept.
9. Criticise existing services or rescue activities in front of people in need of those services.

Actions that are likely to help all people affected by an emergency or incident are to promote a sense of safety, calm, connectedness, self-efficacy and help.

**Recognising and Managing Stress**

When people are exposed to traumatic events, it is necessary to be aware how these events can affect people, including yourself, personally. Most people show signs of stress after such events. When these experiences are upsetting, they may be called distress.

Over time, as life gets back to something that feels like normal, these experiences should decrease.

After a stressful event, people should be encouraged to monitor their own physical and mental health by knowing the signs of stress in themselves, their colleagues and their family and friends and how to get help. We summarise some of the ways this may be achieved.

*Know the Signs of Stress*

**P**eople may find their energy and activity levels may increase or decrease, feel tempted to increase use of tobacco, alcohol or other substances, experience irritability, with outbursts of anger and frequent arguing, have trouble relaxing or sleeping, recurring dreams and nightmares and cry frequently, worry excessively. They may also want to be alone most of the time, experience relationship difficulties, blame other people for everything, have difficulty communicating or listening as well as giving or accepting help. They may be less able to feel pleasure or have fun and experience decreased libido/sexual interest.

People may find that signs of stress manifest physically as, for example, stomach aches or diarrhoea, headaches or other pains, loss of appetite or eating too much, sweating or having chills, having tremors or muscle twitches, parathesis e.g. numbness and tingling in extremities.

People may also feel anxious or fearful, have mood swings, self-doubt, or feel sadness, feel guilty angry, heroic, euphoric or invulnerable. They may lack concern about things that usually interest them and feel overwhelmed by sadness. Also people may have trouble remembering things, have reduced attention span, feel confused, have trouble thinking clearly and concentrating and have difficulty making decisions.

*Know How to Relieve Stress*

**P**eople can be encouraged to take care of themselves and know how they can manage and alleviate their own stress. Methods that have been found helpful include keeping healthy by managing diet and hydration, avoiding excessive amounts of caffeine, tobacco, alcohol or other non-prescribed substances and making sure to get enough sleep and rest and taking physical exercise.

*Practical Ways to Relax*

People can be encouraged to relax themselves by doing things that work for them e.g. walking, football, taking a bath, listening to music, cooking, carrying on with their hobbies. Hard work or stressful tasks should be rewarded by doing fun things afterwards. Talk about feelings to family and friends as often as seems needed. It is important to recognise and take notice of early warning signs of stress, how previous past experiences affect the way of handling other events and know that feeling stressed, depressed, guilty, or angry is common after an emergency event.

**Managing Intense Feelings in Other People**

When people are faced with an emergency and you first meet them, intense feelings are often present and appropriate. They are the result of fear, uncertainty, and apprehension. If the person becomes agitated, he or she may feel that their personal space is being threatened, challenge or question authority, refuse to follow direction, lose control and become verbally agitated and/or become threatening. These suggestions are equally applicable to colleagues, patients, family, friends and community members.

Actions that help staff to manage those intense feelings can include communications calmly using the SOLER technique (Sit squarely; Open Posture; Lean Towards The Other; Eye Contact; Relax) while communicating warmth as a relationship is stablished. It helps to use concrete questions to help people to focus on what the listener may wish to know, to attempt to come to an agreement on something and always to speak to the person with respect.

**ONLINE RESOURCES**

General Medical Council. *The State of Medical Education and Practice in the UK*. GMC, 2018.

British Medical Association. *Caring for the Mental Health of the Medical Workforce*. BMA, 2019.

NHS England & Improvement and Health Education England. *We are the NHS: People Plan for 2020/2021 - action for us all*. NHS England & Improvement, London, 2020. ([www.england.nhs.uk/wp-content/uploads/2020/07/We-Are-The-NHS-Action-For-All-Of-Us-FINAL-March-21.pdf](http://www.england.nhs.uk/wp-content/uploads/2020/07/We-Are-The-NHS-Action-For-All-Of-Us-FINAL-March-21.pdf))

Association of Chief Ambulance Officers. *Supporting ambulance staff on mental health and wellbeing.* Association of Chief Ambulance Officers, 2019. ([aace.org.uk/mentalhealthandwellbeing/](https://aace.org.uk/mentalhealthandwellbeing/))

College of Paramedics. Paramedic mental health and wellbeing: Your mental health. College of Paramedics, 2020. (collegeofparamedics.co.uk/COP/Member_/Paramedic_Mental_Health_and_Wellbeing.aspx)

## College of Paramedics. Guidance for managers on psychosocial support and mental wellbeing of ambulance personnel in a pandemic; 2020. ([collegeofparamedics.co.uk/COP/News/Covid-19/Guidance_for_managers_psychosocial_support_and_mental_wellbeing_of_ambulance_personnel_in_a_pandemic.aspx](https://collegeofparamedics.co.uk/COP/News/Covid-19/Guidance_for_managers_psychosocial_support_and_mental_wellbeing_of_ambulance_personnel_in_a_pandemic.aspx))

The Ambulance Service Charity. Mental Health Support. TASC; 2020. ([www.theasc.org.uk/services-we-offer/mental-health/](http://www.theasc.org.uk/services-we-offer/mental-health/))

Office for Health Improvement and Disparities. *Prevention Concordat for Better Mental Health.* Office for Health Improvement & Disparities, 2023. ([www.gov.uk/government/publications/prevention-concordat-for-better-mental-health-consensus-statement/prevention-concordat-for-better-mental-health](http://www.gov.uk/government/publications/prevention-concordat-for-better-mental-health-consensus-statement/prevention-concordat-for-better-mental-health))

National Fire Chiefs Council. Mental Health and well-being. (www.nationalfirechiefs.org.uk/health-and-well-being)

**GLOSSARY OF TERMS**

| **Term** | **Definition** |
| --- | --- |
| Burnout | A syndrome conceptualised as resulting from chronic workplace stress that has not been successfully managed. It is characterised by three dimensions: feelings of energy, depletion or exhaustion; increased mental distance from one’s job or feelings of negativism or cynicism related to one’s job; and, reduced professional efficacy. |
| Distress | People are likely to feel stressed in emergencies and incidents. Their experiences are described as distress when they are accompanied by emotions, thoughts, and physical sensations that are upsetting or which effect their relationships. Recent research shows that common experiences that people describe as distress include feeling upset; fear; anxiety; fear of recurrence of the event; vigilance at social gatherings and in public places; avoiding uncomfortable feelings; and social withdrawal. The main differences between distress and the symptoms of common mental health problems lies in the trajectory of people’s recovery and the severity of their experiences. Until recently, the literature has tended to underestimate the number of people who take a long time to recover. |
| Emotional labour | Emotional labour involves the suppression of a person’s own feelings. Their outward appearance produces in others, a sense of being cared for in a safe place. It is a key element in ensuring compassionate care. |
| First responder | A person with or without specialised training who is among the first to arrive and provide aid at the scene of an incident or emergency. Most first responders are friends, family members or members of the public; usually, they render invaluable assistance. |
| First professional responder | A person with specialised training who is among the first to arrive and provide aid at the scene of an incident or emergency. |
| Major incident | A major incident is any occurrence that presents serious threat to the health of the community or causes such numbers or types of casualties, as to require special arrangements to be implemented. For the NHS, this includes any event defined as an emergency under the Civil Contingencies Act 2004 |
| Mental healthcare | Formal biomedical and psychological treatments for mental health problems and disorders that are delivered by trained mental health practitioners. Psychosocial care is often required at the same time as a platform for these specialised treatment |
| Moral distress | Moral distress occurs when staff are unable to deliver the level of care they would like, owing to organisational (structural) constraints. for example, failures in leadership, and the types of injury/illness treated in pre-hospital settings, hospitals, and communities, are linked to psychosocial distress in healthcare practitioners. Moral distress arises here because the aspirations of staff to deliver high-quality care are not realised owing to limitations in the quality of care that services are able or willing to support. |
| Moral injury | Moral injury describes the psychological consequences of bearing witness to violence and human carnage and its aftermath. It encompasses witnessing human suffering or failing to prevent outcomes that transgress deeply held beliefs, such as the rights of children to be protected by their parents, or the belief that life can and should be preserved by appropriate and timely medical intervention. It also recognises failings in leadership, where staff are not appropriately resourced whether in terms of people, space, or equipment. |
| Needs and responses | Needs refer to people’s requirements for assistance because of their exposure to an emergency or incident. Responses refers to the ways in which societies, communities, relatives, formal services, and practitioners act to meet the needs of people and communities after major incidents. |
| Post Traumatic Growth | Positive psychological change experienced because of a person’s struggle with challenging life circumstances. This can lead to them revising and developing new psychological and philosophical beliefs. It can stimulate growth across three domains: self-perception, interpersonal relationships, and their philosophy of life. |
| Post-traumatic stress disorder (PTSD) | PTSD can develop after a major incident or other stressful event or situation of an exceptionally threatening or catastrophic nature. It may affect up to 25–30% of people of any age who have experienced an event of this nature. Symptoms include:  • re-experiencing (including nightmares)  • avoidance  • hyperarousal (including hypervigilance, anger and irritability)  • negative alterations in mood and thinking  • emotional numbing  • dissociation  • emotional dysregulation  • interpersonal difficulties or problems in relationships  • negative self-perception (including feeling diminished, defeated or worthless) |
| Primary stressors | Primary stressors are inherent in emergencies. The term describes the sources of worry, anxiety, and stress that stem directly from the events and consequential tasks that the staff of services face during their work. |
| Psychological safety | Psychological safety is one component of organisational and team culture. When present, people believe that others will not resent or penalise them for asking for help, information, or feedback in psychologically safe environments. It avoids blame, belittling and undermining and emphasises constructive learning that has substantial effects on staff wellbeing. |
| Psychosocial | This describes the emotional, cognitive, social, and physical experiences of people in the context of their environments. It describes the interactions between psychological and social processes within and between people and across groups of people. |
| Psychosocial care | Psychosocial care describes interventions for people who are distressed whether their distress is accompanied by thoughts and feelings that interfere with their day-to-day function or if they have symptoms of a mental health problem that do not reach a diagnosis. |
| Stress | Stress is a term that is used widely and often inconsistently. Sometimes it refers to a stimulus (more appropriately described as a stressor) and sometimes to people’s responses. Here, stress describes a collection of common human psychological, physical, and behavioural responses to external and internal challenge. It can be positive when it motivates people but is a problem when the level of stress people experience is overwhelming and unpleasant. Then the experiences are described as distress. Most people experience stress in emergencies and incidents because these events may undermine their positive perceptions of the environment, themselves, their sense of control and feelings of worth. |
| Stressor | An event, circumstance, other occurrence, attitude, response, or something else that stimulates people to experience a stress response, or which causes a state of strain or tension. |
| Validation | People who are affected by emergencies and incidents regard social and professional acknowledgement of their experiences as key to their recovery. This process is called validation. It emphasises the importance of ensuring that opportunities are created for other people, whose opinions are respected, to recognise and acknowledge the experiences of Blue Light staff. |
| Wellbeing | This term is used in this report to refer to every member of staff’s needs for certain sources of support to ensure that they are able to continue to develop, enjoy the stimulation of their work, and flourish. Every member of staff, for example, requires effective leadership and to be a member of a cohesive team that is supporting and nurturing. |

**REFERENCES**

1. Williams R, Kemp V, Batt-Rawden S, Bland L, Burgess J, McInerney A, et al. *Valuing staff, valuing patients: the report on the psychosocial care and mental health programme*. Faculty of Pre-hospital Care, Royal College of Surgeons of Edinburgh, 2021. ([fphc.rcsed.ac.uk/education-resources](https://fphc.rcsed.ac.uk/education-resources))
2. Williams R, Kemp V,. *Psychosocial and mental health care before, during and after emergencies, disasters and major incidents.* In C Sellwood, A Wapling (eds). Health emergency preparedness and response. CABI, 2016.
3. Williams R, Kemp V. Principles for designing and delivering psychosocial and mental healthcare. *BMJ Mil Health* 2020;166(2):105-10. doi:10.1136
4. Patel V. Rethinking mental health care: bridging the credibility gap. *Intervention* 2014;12:15-20.
5. Stevenson D, Farmer P. Thriving at Work: *The Stevenson/Farmer Review of Mental Health and Employers*. Department for Work and Pensions and Department of Health and Social Care; 2017. ([assets.publishing.service.gov.uk/government/uploads/system/uploads/attachment_data/file/658145/thriving-at-work-stevenson-farmer-review.pdf](https://assets.publishing.service.gov.uk/government/uploads/system/uploads/attachment_data/file/658145/thriving-at-work-stevenson-farmer-review.pdf))
6. British Medical Association. *Mental Wellbeing Charter*. BMA, 2020.
7. NICE Guideline [NG116] *Post-traumatic stress disorder*. NICE, 2018.
8. Milligan-Saville JS, Tan L, Gayed A, Barnes C, Madan, Dobson M et al. Workplace mental health training for managers and its effect on sick leave in employees: a cluster randomised controlled trial. *The Lancet Psychiatry* 2017; 4(11):850-858.
9. Williams R, Murray E, Neal A, Kemp V. *The top ten messages for supporting healthcare staff during the COVID-19 pandemic*. Royal College of Psychiatrists, 2020. (top-ten-messages-williams-et-al.pdf)
10. General Medical Council. *Caring for Doctors. Caring for Patients*. GMC, 2019. (www.gmc-uk.org/-/media/documents/caring-for-doctors-caring-for-patients_pdf-80706341.pdf)
